# Neurofibromatosis type I with thoracic involvement

**DOI:** 10.36416/1806-3756/e20260131

**Published:** 2026-08-13

**Authors:** Felipe Von Ranke, Gláucia Zanetti, Edson Marchiori

**Affiliations:** 1. Joaquim Chaves Saúde, Lisboa, Portugal.; 2. Universidade Federal do Rio de Janeiro, Rio de Janeiro, Brasil.

**Figure 1:**
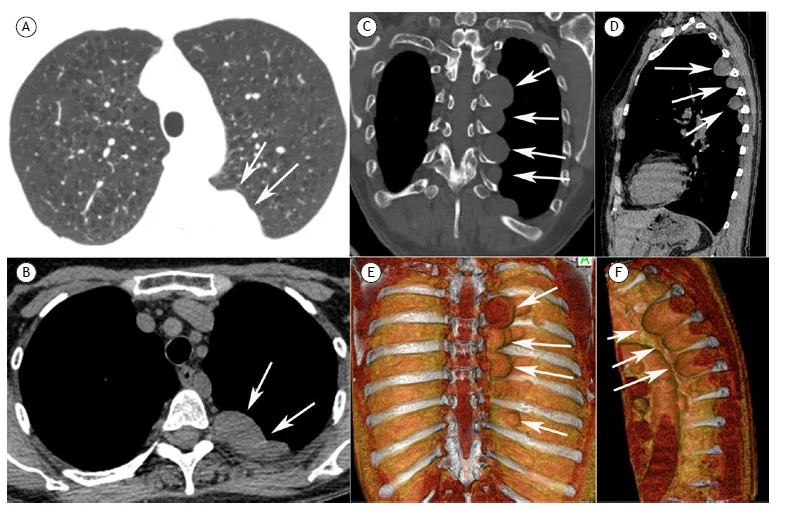
(A) Axial chest CT image showing numerous small, rounded, emphysematous cysts in both lungs. Axial (B), coronal (C), and sagittal (D) reconstructions with mediastinal window settings demonstrating paravertebral and intercostal soft-tissue masses involving the left neural foramina at multiple vertebral levels, with extension along intercostal spaces, following the courses of corresponding thoracic nerve roots and intercostal nerves (arrows). Coronal (E) and sagittal (F) three-dimensional volume-rendered CT reconstructions show the spatial relationships among the masses, vertebral bodies, and posterior ribs.

A 56-year-old non-smoking female with known neurofibromatosis type I (NF1) diagnosed at the age of 15 years presented with chest pain and cough. She denied fever, chills, and other symptoms. The patient had a family history of NF1. Physical examination revealed classical NF1 features, including sparse cutaneous neurofibromas and café-au-lait spots, mainly on the trunk. Chest CT showed emphysematous lung cysts and paravertebral and intercostal soft-tissue masses following the courses of thoracic nerve roots and intercostal nerves, suggestive of neurofibromas (Figure 1). Alpha-1 antitrypsin levels were normal. Biopsy of a paravertebral mass was performed, and histopathology confirmed plexiform neurofibroma. The patient fulfilled the criteria for NF1 and was referred for specialized outpatient follow up. Her course was stable.

NF1, also known as von Recklinghausen’s disease, is an autosomal-dominant inherited neurocutaneous disorder caused mainly by mutations in the *neurofibromin 1* gene. Diagnosis of NF1 is clinical and based on established criteria, requiring the presence of at least two of seven specific features (multiple café-au-lait macules, neurofibromas, axillary or inguinal freckling, optic gliomas, Lisch nodules, osseous lesions, and a first-degree relative with NF1).^1^ ^-^ ^4^ Plexiform neurofibromas, as seen in our patient, are considered to be pathognomonic of NF1.

NF1 can affect the thorax in several ways, including cutaneous and subcutaneous neurofibromas on the chest wall, kyphoscoliosis, intrathoracic neurogenic neoplasms (particularly neurofibromas), meningoceles, and pulmonary manifestations. The most common pulmonary manifestations in NF1 are diffuse interstitial lung disease; pulmonary hypertension; and emphysematous, cystic, and bullous changes.^1^ ^-^ ^4^ An association between NF1 and lung cancer has been described.^2^
